# Computed Tomography versus Optical Scanning: A Comparison of Different Methods of 3D Data Acquisition for Tooth Replication

**DOI:** 10.1155/2019/4985121

**Published:** 2019-04-10

**Authors:** Tomasz Kulczyk, Michał Rychlik, Dorota Lorkiewicz-Muszyńska, Monica Abreu-Głowacka, Agata Czajka-Jakubowska, Agnieszka Przystańska

**Affiliations:** ^1^Section of Dental Radiology, Department of Biomaterials and Experimental Dentistry, Poznań University of Medical Sciences, Poland; ^2^UniversitätsCentrum fur Orthopedie und Unfallchirurgie, Universitätsklinikum Carl Gustav Carus, TU Dresden, Germany; ^3^Department of Virtual Engineering, Poznań University of Technology, Poland; ^4^Department of Forensic Medicine, Poznań University of Medical Sciences, Poland; ^5^Department of Temporomandibular Disorders, Division of Prosthodontics, Poznań University of Medical Sciences, Poland

## Abstract

**Objectives:**

The study aimed to compare the accuracy of different methods of data acquisition and data reconstruction and to assess their usefulness for 3D printing of tooth replicas.

**Methods:**

3-dimensional models of molar and canine teeth obtain utilizing CBCT examination with different protocols, and optical scanning was compared with models derived from micro-computed (micro-CT) examination using Geomagic Studio Qualify software. A pairwise comparison of 3D models with analysis of standard deviation and the value of the mean distance of given surfaces was performed.

**Results:**

Standard deviation and the value of the mean distance were lowest for optical scanning followed by CBC in high and standard resolution in all tested protocols. Models, obtained with high-resolution CBCT protocols, of teeth in and outside of alveolar bone showed similar average distance parameters, but standard deviation parameter was significantly lower for models of teeth scanned outside of the socket. Good surface representation on all models was seen at relatively smooth areas while in areas of high changes in the geometry CBCT based models performed inferiorly to those obtained from an optical scanner.

**Conclusions:**

In case of teeth of noncomplicated texture, independently from a position (within or outside the alveolar socket), the high-resolution CBCT seems to be a sufficient method to obtain data for 3D printed tooth replica. Optical scanning performs better when a detailed replica is necessary.

## 1. Introduction

Three-dimensional (3D) printing is an advanced technology that models object scanning data, introduces it to the device, and controls the precise 3D placement of the printed material in order to create a model similar to the original object [1, 2]. Since 3D printing was first conceptualized and used in the early 1980s [3], its fast development leading to the significant increase in the use of 3D printing in biomedical sciences and medical industry has been observed [3, 4]. Historically, one of the earliest applications of 3D printing in medical modeling was a production of an anatomical study model [5]. Until today, anatomically accurate models and patient's specific models to assist with the planning of complex treatment are most often the subjects of 3D printing, and methods of obtaining objects ideally depicting the original are continually researched [6–8]. The 3D printing offers numerous benefits; therefore its application in many fields, i.e., premedical education, surgery, and medical research, can be observed [4, 5, 9–11].

Likewise, the impact of 3D printing in dentistry is increasingly important. The evolution of 3D printing in the dental sector is mainly due to its availability and low costs; therefore, the digital manufacturing technologies have become firmly established among professionals [12]. In recent years, 3D printing technology has been increasingly used in orthodontics, restorative dentistry, maxillofacial, and implantology [12–14]. Moreover, the usefulness of 3D printing in the fabrication of replica teeth and auto-transplantation has been reported [13–17].

The 3D printing technology has particular resonance with dentistry due to ready access to volumetric data in the form of cone-beam computed tomography (CBCT) and optical scanning data [12]. The design and production of 3D printed objects rely on the exchange of digital data between 3D imaging, 3D virtual planning, and 3D printing technologies [18]. To use the 3D printed dental models for clinical purposes, the accuracy of the printed outcome must be ensured [19]. The creation of the 3D model prior to printing can be done by converting data from different imaging modalities. Currently, physical models can be reproduced from digital data obtained in various ways, but the accuracy of the printed object depends on the input volumetric data. The computer aided design software that allows objects to be designated in a virtual environment is crucial for the 3D printing process [12].

The demand for this research came with the archaeologists' need for complementation of the exhibit's tooth originally taken for genetic investigations. Teeth are a good source of valuable genetic material for identification research. However, in the process of extracting pulp residues for genetic analysis, the tooth is irreversibly destroyed. In the process of documenting the results of identification tests, x-ray examinations are carried out using tridimensional techniques such as CBCT tomography. This study assesses that data collection protocol will be the most appropriate to compile the source material to create replicas. It was decided to investigate whether the x-ray images of teeth in the alveolus would be a sufficiently good source of data for generating models or whether a better way would be to remove them from the alveolus and perform X-ray or optical scanning.

This study focuses on analysis of different methods of data acquisition for further replica formation in terms of their availability to provide sufficient reproduction of details.

Thus, the purpose of the study was to compare the accuracy of three different methods of data acquisition and data reconstruction (standard and high-resolution CBCT and optical scanning) and to assess their usefulness for 3D printing of tooth replicas.

## 2. Materials and Methods

To obtain and compare 3D data of teeth, a number of methods were employed, namely, the CBCT examination, optical scanning, and micro-computed (micro-CT) examination.

### 2.1. CBCT Examination

The lower canine and the first molar located in their anatomical positions in human dry mandible were scanned by means of CBCT unit (Scanora 3D, Soredex, Finland) in two modalities: standard resolution with Field of View (FOV) 13x14.5 cm and voxel size of 0.35 and high resolution with FOV 6x6cm and voxel size of 0.133. During CBCT scanning, the mandible was initially placed in a normal anatomical position with a long axis of the teeth being perpendicular to the occlusal plane. After completing the scanning the teeth were removed from the alveolar sockets and scanned again separately in standard and high resolution with a long axis of teeth at 90 degrees in relation to the occlusal plane. The data obtained from all radiographic modalities was exported as DICOM files for further evaluation.

### 2.2. Optical Scanning

The canine and the first molar were scanned by means of the optical scanner (Ceramill map 400+ Amann Girbach AG, Austria) which operates with the accuracy of 6 *μ*m. This method allows for noncontact data collection from many different directions to cover the whole geometry of the object. After assembly of all measurements from different directions into one object, 3D surface triangle mesh was obtained in STL format with the use of proprietary software distributed with a 3D scanner.

### 2.3. Micro-CT Examination

The canine and the first molar were scanned separately with micro-CT with the resolution of 0.012mm (Nanotom S, GE, USA) and data obtained was exported as DICOM files for further evaluation.

### 2.4. Creating 3D Computer Models Based on CBCT Micro-CT and Optical Scanner Data

Teeth models were created based on DICOM files data obtained from CBCT and micro-CT examination, and from data provided by the proprietary software from the manufacturer of the optical scanner.

In each of the scans sets, the areas of the tooth have to be marked in the segmentation process. Segmentation was done by an algorithm of the ScanIP software (Simpleware) where, in the final step, the high accuracy triangle surface mesh of every tooth in every modality was created. As the output, the 3D models were exported in the STL format.

The 3D model obtained from micro-CT—due to its highest currently available quality (resolution)—served as a gold standard for evaluation and comparison for other models. Summarized input and output data used for 3D model creation are presented in Table 1.

### 2.5. The Model Comparison Procedure

A pairwise comparison of obtained 3D models (in STL format) was performed with the micro-CT model serving as a reference model. Comparison analysis between models was performed by an automatic algorithm implemented in Geomagic Studio Qualify software, which is a standard tool into technical comparisons of 3D models (Computer Aided Design systems). For a correct implementation of the comparative process, it was necessary to overlap individual models so that they occupy exactly the same position in 3D space. This so-called registration process, performed for all models, was made in two stages in the specialized reverse engineering software—Geomagic Studio. As a base for positioning tooth models in 3D space, a model obtained from micro-CT was used (with the* z*-axis oriented along the vertical tooth line). In the first stage, a manual multi-point registration was performed. For this purpose, a set of 9 characteristic, unique points on the surface of the models, including top of apices and crowns, as well as points determining the border of enamel and dentine, were chosen, then an automatic matching process whose algorithm relies on minimizing the distance (mean square error) between two objects. The procedure for registering 3D objects used in the paper is a standard solution commonly used in reverse engineering.

Finally, prepared models were then subjected to a geometry comparison analysis between the models. The analysis was performed in the Geomagic Studio Qualify software. The analyzed parameters between surfaces of the models 3D models were maximum distance (minimum and maximum), average distance (minimum and maximum), and standard deviation. Additionally, the graphical representation (graphical map) of distortion projected onto surface 3D model was created for every compared pair of models.

## 3. Results

**Figure 1:**
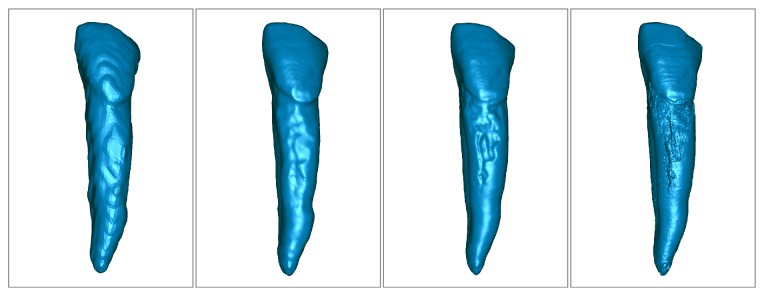
Visualization of 3D models obtained in different methods: CBCT in standard and high resolution, optical scanner, and micro-CT.

 A sample visualization of 3D models of canine tooth obtained with different techniques was presented in Figure 1. The difference in the quality of 3D models obtained in the different methods can be observed. As expected, the lowest quality is presented by the model obtained from CBCT with standard resolution. In addition to the loss of many details of the tooth, certain bands (layers) are visible, resulting from a small number of slices used for model creation. In the case of a model obtained from CBCT high resolution, the band structure of the model is not visible and some previously invisible details appear. The model developed on the basis of data from an optical scanner has more distinct edges of the boundaries of areas, as well as an increased level of reproduction of finer details. The micro-CT model shows the highest quality with precise information about the rigid structure of the root surface.

**Figure 2:**
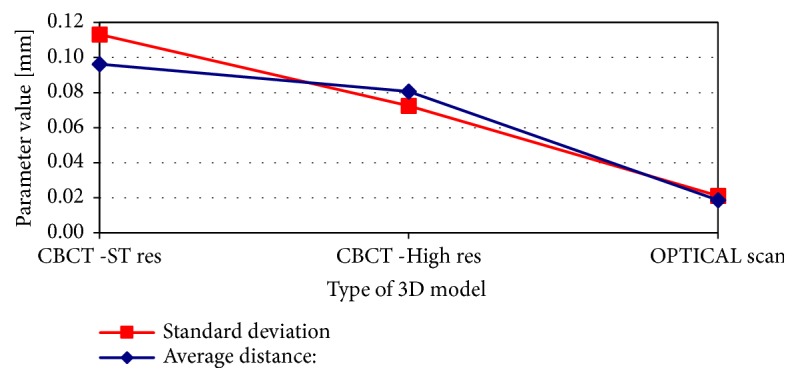
Pair-wise comparison of 3D models of molar tooth created in CBCT standard resolution, CBCT high resolution, and optical scanner against reference micro-CT 3D model.

**Figure 3:**
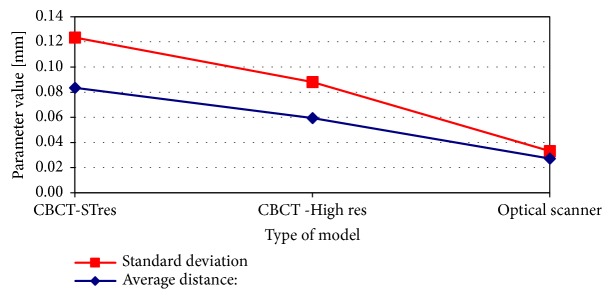
Pair-wise comparison of 3D models of canine tooth created in CBCT standard resolution, CBCT high resolution, and optical scanner against reference micro-CT 3D model.

Results of pairwise comparative analysis of 3D models in relation to the “golden standard” micro-CT models are presented in Figures 2 and 3. Analysis of the parameters obtained in the comparison procedure of 3D models confirmed earlier visual observations. Both the standard deviation and the value of the mean distance decrease with the increase of the quality of the model (scan accuracy). In both cases, the teeth of the molar and the canine, the best results (lowest level of parameters values) were achieved for the models obtained on the basis of optical scanning. The 3D model based on the optical scanner is 10 times more detailed in terms of the number of triangles describing the tooth surface and 4-5 times more accurate in terms of standard deviation, in comparison to the model obtained from the standard resolution of CBCT. This increase in accuracy is also reflected in the number of triangles describing the surface of the 3D models. In the case of a model from an optical scanner, the number of triangles is about 10 times higher than in the case of a standard CBCT and about 1/3 greater in comparison to the model obtained from high-resolution CBCT.

**Figure 4:**
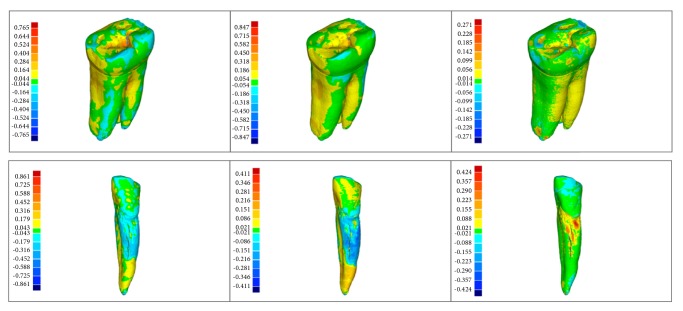
Distortion projected onto surface of micro-CT 3D molar and canine teeth model of CBCT in standard, high resolution, and optical scanner.

A graphical representation of distortion projected onto the surface of micro-CT 3D molar and canine teeth model of CBCT in standard, high resolution, and optical scanner, respectively, are presented on Figure 4. Green color represents the best correlation between the surface of the analyzed and reference model, red color the maximum (plus value) distortion between models, and blue color the minimum (minus value) between models. Generally, good surface representation in models is visible for areas with so-called low dynamics of geometry changes (i.e., relatively smooth areas). The case of areas of high dynamics of changes in the geometry (an area rich in fine detail) shows the inferior image original shape and the “lost" in this way, many details of the tooth.

**Figure 5:**
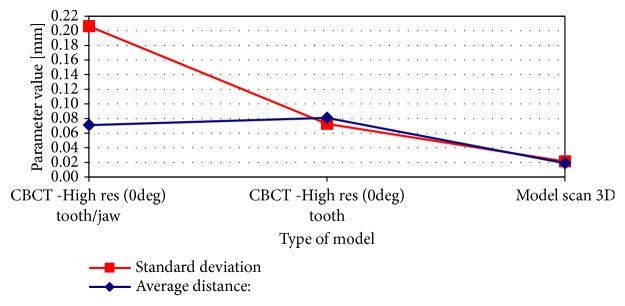
Pair-wise comparison of 3D models of molar tooth created in CBCT High resolution with tooth in the bone, CBCT high resolution, and optical scanner against reference micro-CT 3D model.

**Figure 6:**
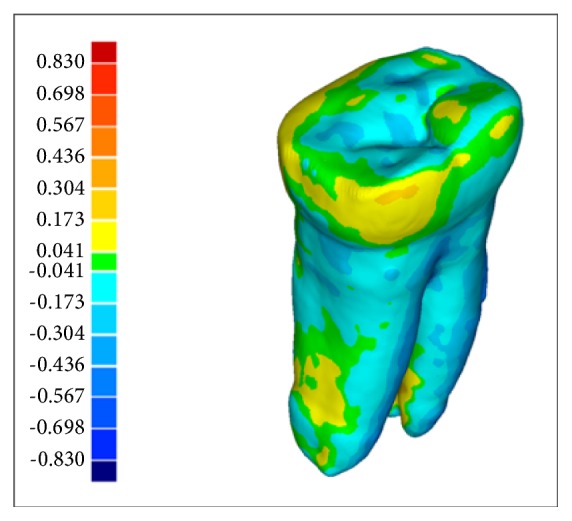
Distortion projected onto surface of 3D model molar tooth in CBCT High resolution and CBCT high resolution of tooth in the bone.

In addition, a comparative analysis for the reconstruction of a tooth model made on the basis of the high-accuracy CBCT model made for a tooth in the jaw and the same tooth removed from the jaw was performed. The obtained results presented in Figures 5 and 6 showed that for the average distance parameters there was not a big difference between the given models, but, in the case of the standard deviation parameter, the difference in the results is significant.

## 4. Discussion

One cannot disagree with the fact that the accuracy of objects made by 3D printing will be influenced by the technology applied [13]. According to the review by Martelli et al. [8], every fifth study stressed that the accuracy of the objects obtained by 3D printing techniques was not always satisfactory. This could be attributed to the resolution of the 3D images used to model the printed objects. It is considered, that, by choosing the more professional or industrial printer, 3D replicas are more realistic and precise [9]; however, the process of making medical models involves various steps, each of which can be a source of error. Therefore, errors can occur during imaging, image segmentation, or 3D printing phase [20]. Throughout the process, from medical imaging to the 3D printed anatomical models, the choice of an appropriate image segmentation algorithm is arguably the most important step [6]. The creation of the 3D model to be printed can be done through various types of computer aided design software, or by converting data from imaging modalities. In the case of medical modelling, original imaging modality will often be the limiting factor of accuracy [20]. The higher the resolution of the original data, the better the quality of the 3D model and the better the quality of the 3D printed object.

The CBCT imaging techniques commonly used in medicine have also become a method of data completion, which are used to later obtain a 3D reconstruction of anatomical structures.

CBCT imaging is achieved by using a rotating gantry with an x-ray source and detector. A cone-shaped source of radiation is directed through the middle of the area of interest onto an area x-ray detector on the opposite side. The x-ray source and detector rotate around a rotation fulcrum fixed within the center of the region of interest. Because CBCT exposure incorporates the entire FOV, only one rotational sequence of the gantry is necessary to acquire enough data for image reconstruction. During the rotation, multiple sequential planar projection images of the field of view (FOV) are received [21]

Practically, some factors that influence the CBCT image quality (i.e., scanning data and reconstruction parameters) may also influence the quality of 3D models reconstructed from CBCT images [22, 23].

In the present study, we decided to use a 3D reconstruction obtained by micro-computed tomography (micro-CT) as the comparative model, because it enables imaging of essential anatomical elements with ultra-high accuracy (in comparison to other sources). The micro-CT provides 3D digital datasets with comparable resolution to light microscopy [24]. Its precision and usefulness in the manufacturing of anatomical details with 3D printing were confirmed by Kuru et al. [7], who were able to reproduce the functioning anatomical middle ear model which was segmented from micro-CT data. Shelmerdine et al. [24] reported the usefulness of 3D printed micro-CT imaged specimens for craniofacial surgical applications, developmental cardiac anatomy, placental imaging, archaeological remains, and high-resolution bone imaging. It was also stated that micro-CT allows for detailed investigation of teeth and volumetry of root canals [25, 26]. This imaging method also has some limitations, i.e., small sample size, in vitro conditions, long time requirements, and high costs involved, which in some cases can be impossible to overcome. An additional disadvantage of micro-CT is a large number of slices which causes a significant demand for the computing power of the computer, and, in extreme cases (especially for larger object sizes), it can even prevent the reconstruction of the 3D model of the analysed object.

The presented study showed that neither optical scanning nor the CBCT equals the micro-CT. Nonetheless, high-resolution CBCT may serve as 3D data sources if the process has to be accelerated or if it is not necessary for the 3D printed object to be so accurate.

The micro-CT scans have higher image resolution, with the voxel size less than 0.001mm compared with 0.113 for CBCT.

Moreover, 3D models created on the basis of micro-CT data provide significantly better accuracy precision than 3D printers and CNC machines are able to produce the replicas with it.

Van Dessel et al. [27] demonstrated that most CBCT scanners may be capable of quantitative analysis of the bone with a level of accuracy and reliability that approaches micro-CT. Maret et al. [28] assessed the accuracy of CBCT units by comparing volumetric measurements reconstructed with two isotopic voxel sizes against the references of smaller voxel size CBCT and micro-CT and reported that volumetric measurements made with CBCT are all similar for voxel sizes up to 200 *μ*m, despite a slight tendency towards underestimation. At 300 *μ*m and above the underestimation of the measurements becomes statistically significant.

The accuracy of metric characteristics of digitally created 3D reconstructions of anatomical structures has been previously studied. It has been proved that linear and angular measurements of 3D models based on both laser scanning and CT scans are reliable and accurately correspond to the original bones [13, 29–31]. Following these conclusions, it can be believed that an object printed on the base of 3D digital reconstruction should also ideally depict the original.

Although our study revealed minor differences between dataset created by CBCT and optical scanning, we agree with results of Kamburoğlu et al. [22] who assessed the reliability of measurements performed on the 3D virtual models obtained using CBCT and 3D optical scanning and stated that the 3D scanner measurements were more accurate. Probably this is why Lee et al. [13] considered statistically significant differences between original teeth and replica teeth made by printing technology based on optical scanning to be clinically acceptable. On the other hand, Naumovich et al. [32] are convinced that modern CBCT scanners do not allow for a creation of perfect replica teeth without distortion.

We believe that three-dimensional models obtained using the CBCT technique can also be used to produce replicas for the training of cavity preparation, where a moderate level of detail in surface mapping is sufficient. Also, 3D printing technology allows differentiating the texture and color of individual tissues forming teeth which can help to create more realistic scenarios for training. To complement the study, the replica teeth have been manufactured using two types of devices: 3D printer and numerically controlled milling machine (CNC). The process of comparing will be continued.

## 5. Conclusions

Significant differences were found between models created with different techniques in the regions of teeth with complicated morphology. In case of teeth of noncomplicated texture, independently from a position (within or outside the alveolar socket), the high-resolution CBCT seems to be a sufficient method to obtain data for 3D printed tooth replica. Optical scanning performs better when a detailed replica is necessary. Regardless of the data acquisition technique, it should also be remembered that the procedure of data processing from a series of slices or measurement data from an optical scanner (or other 3D scanners) also has a significant impact on the obtained 3D model and can lead to errors.

## Figures and Tables

**Table 1:** Input (number of DICOM slices) and output (number of triangles) data used for 3D models creation.

		CBCT standard resolution	CBCT high resolution	Optical 3D scanning	Micro-CT
Molar	Number of slices	71	186	-	2056
	Number of triangles	13 780	98 624	132 924	24 974 112

Canine	Number of slices	85	239	-	2262
	Number of triangles	9 612	65 340	99 042	11 510 788

## Data Availability

Data are available upon reader's request.
